# Tenosynovial giant cell tumor of cruciate ligament: A case report and review

**DOI:** 10.1016/j.ijscr.2022.106771

**Published:** 2022-01-15

**Authors:** Nam Vu Tu, Nang Vo Sy Quyen, Minh Ho Ngoc, Hieu Pham Trung, Ba Son Nguyen, Dung Tran Trung

**Affiliations:** a VinUniversity, College of Health Sciences, Viet Nam; b Vinmec Healthcare System, Orthopaedic & Sports Medicine Center, Viet Nam

**Keywords:** Case report, Tenosynovial giant cell tumor, Cruciate ligament

## Abstract

**Introduction:**

Tenosynovial giant cell tumor (TSGCT) is a slow-growing soft tissue tumor that develops from the synovial tissue of tendon sheaths, joints, or bursae. In the knee, this type of tumor is uncommon. Giant cell tumors of the cruciate ligaments' tendon sheath are far more infrequent. Only 16 localized TSGCTs of the cruciate ligaments have been recorded in the literature to our knowledge, with 9 involving the anterior cruciate ligament (ACL) and 7 involving the posterior cruciate ligament (PCL).

**Case presentation:**

We present a rare case of localized TSGCT arising from the PCL's femoral insertion in a 44-year-old male, as well as a literature review on localized TSGCT of cruciate ligaments in the knee. The diagnosis of tumor's presence was made using MRI while the definitive diagnosis was obtained through intraoperative evaluation and postoperative pathology.

**Conclusion:**

Arthroscopic tumor resection was an effective and safe treatment option based on the available data.

## Highlights

• Giant cell tumors of the cruciate ligaments are extremely uncommon.
• The most typical clinical presentations are pain and decreased range of motion of the knee without a history of trauma.
• MRI helps confirm the presence of a tumor but not its original location or type.
• Based on available data, arthroscopic tumor removal is an effective and safe treatment option.

## 1 Introduction

TSGCT is a soft tissue tumor that develops locally in the synovial tissue of tendon sheaths, joints, or bursae. This type of tumor is quite uncommon in the knee [1]. TSGCT that develops locally from the cruciate ligament is much more uncommon. Only 16 cases of localized TSGCT of the cruciate ligament have been reported in the English literature, including 9 cases of the ACL [[2], [3], [4], [5], [6], [7], [8]] and 7 cases of the PCL [6,[9], [10], [11], [12], [13], [14]]. Otsuka [3] described the first case of TSGCT originating from ACL. Sheppard [10] described the first case of TSGCT arising from PCL. Due to the rarity of this condition, there are no guidelines for diagnosis, treatment, or postoperative follow-up and evaluation. We describe a case of TSGCT of the PCL that we initially mistook for a Cyclops lesion. This case report has been reported in line with the SCARE Criteria [15].

## 2 Case presentation

**Fig. 1:**
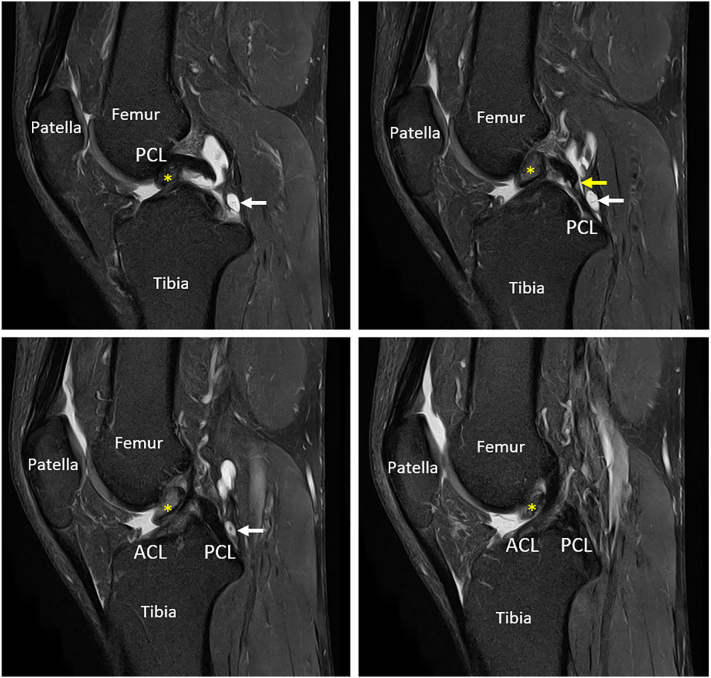
The tumor on sagittal plane MRI of the patient's left knee. Yellow asterisk: tumor; yellow arrow: neglected injury of PCL; white arrow: cyst of PCL; ACL: anterior cruciate ligament; PCL: posterior cruciate ligament.

A 44-year-old male patient complained of discomfort in the left knee while extending for two months. The pain began spontaneously and was vague. The patient previously underwent conservative treatment for a grade II PCL injury ten years ago. Clinical examination revealed that the range of motion of the knee was normal. Prior to the onset of the pain, the patient was able to participate in amateur basketball without incident. The patient's MRI revealed a 1.8 × 1.2 × 1.0 cm mass in the intercondylar area, located anterior to the ACL but originating from the PCL's femoral insertion. Additionally, the MRI showed an earlier PCL injury with a synovial cyst behind it (Fig. 1).

**Fig. 2:**
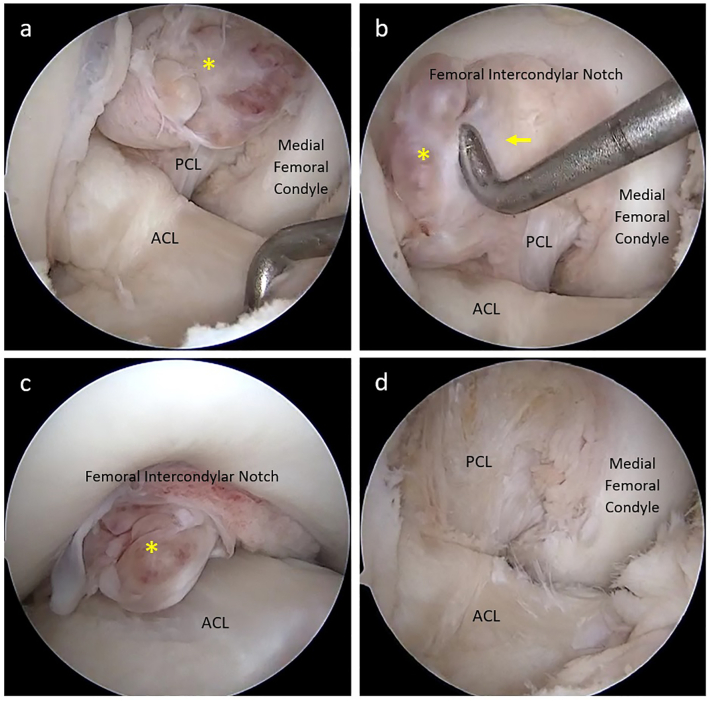
Intra-operative arthroscopic imagines. Yellow asterisk: the tumor; a-ACL; b-the tumor blended with PCL's s membrane; c-the tumor being trapped between intercondylar notch and ACL; d-after the excision.

**Fig. 3:**
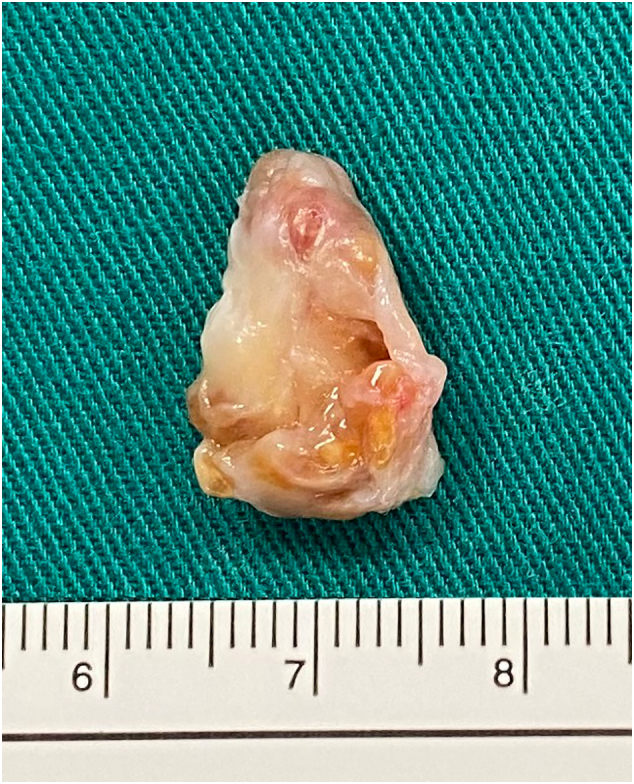
Gross examination of the tumor mass: yellowish, pinkish, and partially shiny white, sizing 1.8 × 1.2 × 1 cm.

**Fig. 4:**
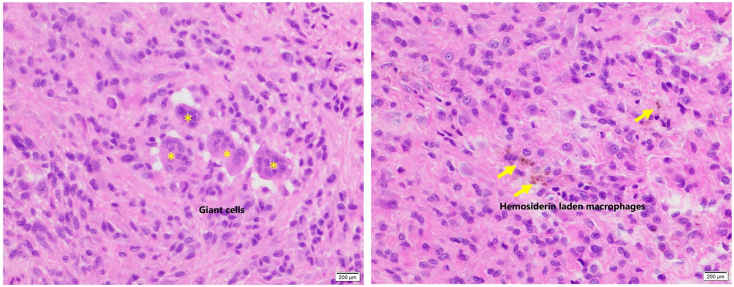
The histopathology of H & E staining (×400) showed scattered multinucleated giant cells (yellow asterisks) and hemosiderin laden macrophages (yellow arrows).

At first, we believed that the mass was fibrous tissue caused by PCL injury. However, endoscopic examination revealed a yellowish, pinkish, and partially gleaming white tumor that was continuous with the synovial membrane at the PCL's femoral origin. The tumor became trapped between the ACL and the intercondylar notch during extension (Fig. 2). The PCL was stretched, impairing its function. By extending the anteromedial portal, we were able to detach the tumor from the PCL and entirely remove it by using a radiofrequency device. The tumor was measured to be 1.8 × 1.2 × 1 cm in size, which was consistent with the size measured on MRI (Fig. 3). The superficial synovial membrane of the PCL was completely removed along with the tumor, leaving only the normal fibrous structure. We made no more interventions. The histopathology findings verified the characteristic lesions of TSCTG (Fig. 4). Following surgery, the patient was promptly admitted to rehabilitation and did not receive radiation therapy. After three months, the range of motion of the knee was fully normal, and the patient's pain disappeared.

## 3 Discussion

TSGCTs are benign, slow-growing tumors that account for approximately 1.6% of soft tissue tumors. They are most frequently seen in the hands (76% of cases), followed by the feet (12%). Only 4% of cases occur in the knee. TSGCTs occur from 4 to 80 years old but are most prevalent between the ages of 30 and 40, with a female/male ratio of around 2:1 [1]. TSGCT of the cruciate ligaments are exceedingly unusual while they have been documented in only a few cases. Gülenç et al. [5] reported the largest series of intra-articular TSGCT of the knee, with seven patients, of which 3 cases originated from the anterior cruciate ligament. Flevas et al. [6] described two cases, one with ACL and one with PCL. Besides the cruciate ligaments, intra-articular TSGCT of the knee can also originate from the fat pad [16,17], the medial plicae [18], the anterior horn of the medial meniscus [19], the lateral retinaculum, the patellar tendon [5], and the ligamentum membrane [20]. We only found 16 cases of TSGCT of the cruciate ligaments reported in literature, comprising 9 cases of ACL and 7 cases of PCL. The average age of these 16 cases was 32 years (18–54), with 7 women and 9 men involved. Four of the thirteen cases with information happened in the left knee, while nine occurred in the right knee.

TSGCTs of the hand or foot are often asymptomatic [1]. However, the most typical clinical presentations of TSGCT of the cruciate ligaments are pain and decreased range of motion of the knee, frequently without an obvious history of trauma. In 16 TSGCTs of the cruciate ligament, except for three cases in Gülenç's report [5] for which data were not available, all remaining patients experienced pain, with 50% having limited range of motion (Table 1). The average time between symptom onset and operation was 2 years (0–5 years). Over 60% of cases were found six months after initiation. Only two cases in Flevas' report [6] were diagnosed immediately after the beginning of symptoms. Only 1/16 of reported cases had a two-month prior history of trauma, and that injury also triggered the pain [10]. Clinical evaluation of all reported cases revealed no evidence of ligament or meniscus injury. In our case, the patient experiences a popping sound when gradually extending the knee from 20 to 30 degrees to full extension, in addition to the pain. We decided to perform endoscopic excision of the lesion without reconstruction of the PCL because the patient was still physically active and had not complained of any other issues before the onset.

**Table 1:** Review of localized TSGCT of cruciate ligaments.

Main author	Year	Age	Gender	Ligament	Side	Time of onset	History of trauma	Pain	Restrict of motion	Injury of ligament/meniscus	Biopsy	Size (cm)	Arthroscopic removal	Follow up	Recurrence
Otsuka	1996	33	F	ACL	Right	6 mos	No	Yes	5°–120°	No	No	2.5 × 2.0 × 0.7	Yes	7 mos	No
Sheppard	1998	47	F	PCL	Right	2 mos	Yes	Yes	No	No	Yes	2 × 2	No (open)	N/A	No
Kim	2003	28	M	PCL	Left	5 yrs	N/A	Yes	No	No	No	1.0 × 1.0 × 0.8	Yes	2 yrs	No
Aksoy	2009	54	F	PCL	Right	2 yrs	No	Yes	No	No	No	2.2 × 1.8	Yes	3 yrs	No
Camillieri	2012	18	M	PCL	Right	2 yrs	No	Yes	No	No	No	4.8 × 2.1 × 2.7	Yes	2 yrs	No
Lee	2014	29	M	ACL	Right	8 yrs	No	Yes	No	No	No	2.0 × 1.0 × 1.7	Yes	4.5 yrs	No
Agarwala	2015	27	M	ACL	Left	3 yrs	No	Yes	No	No	No	1.7 × 0.8 × 0.7	Yes	6 mos	No
Xu	2015	40	F	PCL	Left	2 wks	Yes	Yes	Locking	No	No	2.0 × 1.1	Yes	N/A	N/A
Sayed	2015	38	M	PCL	Right	3 mos	No	Yes	Flx (100°)	No	No	2 × 3 × 2	No (open)	2 yrs	No
Wong	2017	30	F	ACL	Left	1.5 yrs	No	Yes	No	No	No	N/A	No (open)	6 mos	No
Gülenç	2018	32	F	ACL	N/A	N/A	N/A	N/A	Locking	No	Yes	1 × 1	Yes	13 mos	No
		31	F	ACL	N/A	N/A	N/A	N/A	Locking	No	Yes	2.9 × 3	Yes	10 mos	No
		24	M	ACL	N/A	N/A	N/A	N/A	Locking	No	Yes	1 × 1.4	Yes	11 mos	No
Alkhatib	2020	21	M	ACL	Right	4 yrs	No	Yes	No	No	No	1.5 × 1.0	Yes	N/A	N/A
Flevas	2021	32	M	ACL	Right	0	No	Yes	Ext (15°)	No	No	N/A	Yes	3 yrs	No
		26	M	PCL	Right	0	No	Yes	Ext (20°)	No	No	1.3 × 1.9	Yes	6 mos	No

M: male; F: female; ACL: anterior cruciate ligament; PCL: posterior cruciate ligament; Ext: extension; Flx: flexion; N/A: non available; mos: months; yrs: years; wks: weeks.

According to the literature, MRI is the primary approach for confirming the diagnosis of a tumor in the knee joint. TSGCT of the cruciate ligaments could only be definitively diagnosed based on intraoperative evaluation and postoperative pathology. Except for three cases for which data were unavailable, all the remaining cases were undetected on knee X-rays and regular laboratory tests (Table 1). In all cases, the MRI revealed visible masses. Due to the rarity of TSGCT of the cruciate ligament, its characteristics on MRI have not been well studied, and the MRI is only effective for confirming the presence of a tumor, but not for determining the exact original location or type of tumor. There were 4 of 16 patients had preoperative diagnostic biopsies. The remaining authors established a clear diagnosis based on their examination of the tumor's origin during surgery and postoperative pathology. The tumor's size varies from 1 to 5 cm [12,13]. The size of our patient's tumor was 1.8 × 1.2 × 1 cm.

Arthroscopic tumor removal is the favorite treatment option for TSGCTs located in the cruciate ligament. According to previous reports, 13/16 patients had endoscopic tumor removal, while only three cases underwent open surgery, including two cases of PCL adjacent to the posterior capsule [10,11] and one case of ACL. Most of the tumors were located in the fat pad [2]. Although no additional adjuvant treatment such as radiation therapy was given following surgery, the authors observed no recurrence in either open or endoscopic surgery cases. Although the total recurrence rate of TSGCTs varied between 7 and 29% [21], no recurrence was seen at follow-up ranging from 6 months to 4.5 years in 16 reported cases (Table 1). Our patient has only been observed for three months after surgery, however, we believe, like Xu et al. [9], that there will be no recurrence once the tumor has been entirely excised by endoscopy. Our patient will be re-examined with MRI every six months for the first two years. In case of relapse, reintervention by endoscopic surgery could be considered.

## 4 Conclusion

Localized TSGCT of the cruciate ligaments of the knee are extremely uncommon and have received insufficient attention. The primary signs are pain and limited range of motion. The diagnosis of the tumor's presence is made using MRI while a definitive diagnosis is obtained through intraoperative evaluation and postoperative pathology. Based on the available data, arthroscopic tumor resection is an effective and safe treatment option.

## Consent

Written informed consent was obtained from the patient for publication of this case report and accompanying images. A copy of the written consent is available for review by the Editor-in-Chief of this journal on request.

## Provenance and peer review

Not commissioned, externally peer-reviewed.

## Ethical approval

The procedures used in this study inhere to the tenets of the Declarations of Helsinki.

## Funding

We declare no funding for this study.

## Guarantor

Professor Dung Tran Trung MD, PhD.

## Research registration number

This is a case report, so I do not have to register.

## CRediT authorship contribution statement

- NVT contributed to perform the operation
- DTT contributed to revising, and approval for publishing.
- NVSQ contributed to manuscript drafting.
- MHN, HPT contributed to assist the operation, data collection, analysis and interpretation, manuscript drafting.

## Declaration of competing interest

We declare that we have no known competing financial interests or personal relationships with anyone that could have appeared to influence the work reported in this paper.

## References

[1] Ushijima M., Hashimoto H., Tsuneyoshi M., Enjoji M. (1986). Giant cell tumor of the tendon sheath (nodular tenosynovitis). A study of 207 cases to compare the large joint group with the common digit group. Cancer.

[2] Wong J.K., Chan W.H. (2017). Giant cell tumor of the tendon sheath arising from anterior cruciate ligament. New Horiz. Clin. Case Rep..

[3] Otsuka Y., Mizuta H., Nakamura E., Kudo S., Inoue S., Takagi K. (1996). Tenosynovial giant-cell tumor arising from the anterior cruciate ligament of the knee. Arthroscopy.

[4] Lee J.H., Wang S.I. (2014). A tenosynovial giant cell tumor arising from femoral attachment of the anterior cruciate ligament. Clin. Orthop. Surg..

[5] Gulenc B., Kuyucu E., Yalcin S., Cakir A., Bulbul A.M. (2018). Arthroscopic excision of tendinous giant cell tumors causing locking in the knee joint. Acta Chir. Orthop. Traumatol. Cechoslov..

[6] Flevas D.A., Karagiannis A.A., Patsea E.D., Kontogeorgakos V.A., Chouliaras V.T. (2021). Arthroscopic removal of tenosynovial giant-cell tumors of the cruciate ligaments. Presentation of two cases. J. Orthop. Case Rep..

[7] Agarwala S., Agrawal P., Moonot P., Sobti A. (2015). A rare case of giant cell tumour arising from anterior cruciate ligament: its diagnosis and management. J. Clin. Orthop. Trauma.

[8] Alkhatib L.R.K., Sigman Scott A., Stahl U. (2020). Tenosynovial giant cell tumor in the tibial attachment of the anterior cruciate ligament: a case report. Surg. Case Rep..

[9] Xu Z., Mao P., Chen D. (2015). Tenosynovial giant cell tumor arising from the posterior cruciate ligament: a case report and literature review. Int. J. Clin. Exp. Pathol..

[10] Sheppard D.G., Kim E.E., Yasko A.W., Ayala A. (1998). Giant-cell tumor of the tendon sheath arising from the posterior cruciate ligament of the knee: a case report and review of the literature. Clin. Imaging.

[11] Sayed W., Daghfous E., Ben Salah M. (2015). Intra-articular tenosynovial giant cell tumor arising from the posterior cruciate ligament. La Tunisie medicale.

[12] Kim R.S., Lee J.Y., Lee K.Y. (2003). Localized pigmented villonodular synovitis attached to the posterior cruciate ligament of the knee. Arthroscopy.

[13] Camillieri G., Di Sanzo V., Ferretti M., Calderaro C., Calvisi V. (2012). Intra-articular tenosynovial giant cell tumor arising from the posterior cruciate ligament. Orthopedics.

[14] Aksoy B., Erturer E., Toker S., Seckin F., Sener B. (2009). Tenosynovial giant cell tumour of the posterior cruciate ligament and its arthroscopic treatment. Singap. Med. J..

[15] Agha R.A., Franchi T., Sohrabi C., Mathew G., Kerwan A., Group S (2020). The SCARE 2020 Guideline: updating consensus Surgical CAse REport (SCARE) guidelines. Int. J. Surg..

[16] Lucas D.R. (2012). Tenosynovial giant cell tumor: case report and review. Arch. Pathol. Lab. Med..

[17] Abdullah A., Abdullah S., Haflah N.H., Ibrahim S. (2010). Giant cell tumor of the tendon sheath in the knee of an 11-year-old girl. J. Chin. Med. Assoc..

[18] Kim Y.M., Joo Y.B. (2014). Localized nodular tenosynovitis originated near the medial plicae. Knee Surg. Relat. Res..

[19] Kim S.J., Choi N.H., Lee S.C. (1995). Tenosynovial giant-cell tumor in the knee joint. Arthroscopy.

[20] Arican M., Turhan Y., Gamsizkan M. (2020). A rare localized giant cell tumor of the tendon sheath originating from the ligamentum mucosum: a case report. Jt. Dis. Relat. Surg..

[21] Al-Qattan M.M. (2001). Giant cell tumours of tendon sheath: classification and recurrence rate. J. Hand Surg. (Br.).

